# Exploring the enzymatic repertoires of *Bacteria* and *Archaea* and their associations with metabolic maps

**DOI:** 10.1007/s42770-024-01462-3

**Published:** 2024-07-25

**Authors:** Silvia Tenorio-Salgado, José Luis Villalpando-Aguilar, Rafael Hernandez-Guerrero, Augusto César Poot-Hernández, Ernesto Perez-Rueda

**Affiliations:** 1 Instituto de Investigaciones en Matemáticas Aplicadas y en Sistemas, Universidad Nacional Autónoma de México, Unidad Académica del Estado de Yucatán, Mérida, Yucatán México; 2 Unidad de Bioinformática y Manejo de la Información. Instituto de Fisiología Celular, Universidad Nacional Autónoma de México, Coyoacán, Ciudad de México México; 3 Tecnológico Nacional de México, Instituto Tecnológico de Mérida, Av. Tecnológico km. 4.5, 97118 Merida, Yucatan Mexico; 4 Facultad Ciencias de la Salud, Universidad Vizcaya de las Américas, Prolongación Allende, Campeche 24035 Campeche, Mexico

**Keywords:** Metabolism, Enzymes, Allosterism, Comparative genomics

## Abstract

The evolution, survival, and adaptation of microbes are consequences of gene duplication, acquisition, and divergence in response to environmental challenges. In this context, enzymes play a central role in the evolution of organisms, because they are fundamental in cell metabolism. Here, we analyzed the enzymatic repertoire in 6,467 microbial genomes, including their abundances, and their associations with metabolic maps. We found that the enzymes follow a power-law distribution, in relation to the genome sizes. Therefore, we evaluated the total proportion enzymatic classes in relation to the genomes, identifying a descending-order proportion: transferases (EC:2.-), hydrolases (EC:3.-), oxidoreductases (EC:1.-), ligases (EC:6.-), lyases (EC:4.-), isomerases (EC:5.-), and translocases (EC:7-.). In addition, we identified a preferential use of enzymatic classes in metabolism pathways for xenobiotics, cofactors and vitamins, carbohydrates, amino acids, glycans, and energy. Therefore, this analysis provides clues about the functional constraints associated with the enzymatic repertoire of functions in *Bacteria* and *Archaea*.

**Supplementary Information:**

The online version contains supplementary material available at 10.1007/s42770-024-01462-3.

## Introduction

Thanks to the advances of DNA sequencing technology and interdisciplinary approaches in research, the omics era has increased the quantity of genomic information of multiple organisms from the three domains of life. To date, thousands of complete genomes have been deposited in the NCBI portal and are available to the scientific community. In this regard, the large number of experimental data associated with metabolic pathways has opened the possibility to organize and construct databases, such as KEGG [1] and MetaCyc [2], to enable comprehensive and systematic analyses of the adaptive processes of cellular life, the diversity of cellular organization, and the complexities of cellular systems [3].

In this context, the evolution of metabolic pathways can be explained by two general models, mainly based on gene duplication, followed by divergence. The first hypothesis suggests that when a substrate tends to be depleted, gene duplication provides an enzyme capable of supplying the absent substrate, giving rise to homologous enzymes that catalyze consecutive reactions. This scenario has been described in the *Stepwise* hypothesis [4]. Alternatively, duplication of genes encoding promiscuous enzymes (capable of catalyzing multiple reactions) allows each descendant enzyme to specialize in one ancestral reaction, as proposed by the *Patchwork* scenario [5]. Therefore, it is plausible that in the early stages of metabolic evolution, a small number of specialist enzymes existed. Genes encoding these enzymes would have been duplicated, generating enzymes that, through sequence divergence, became more specialized [6].

To date, the metabolic pathway information for a large number of organisms organized into the KEGG database [1] provides an excellent opportunity to make comparative analyses to identify probable enzyme recruitment and duplication events. In this regard, metabolic pathways exhibit high retention of duplicated enzymes within functional modules and coupling of biochemical reactions [7–10].

In this work, we studied the abundance and distribution of the enzymatic reactions in the seven enzyme classes (Enzymatic Commission [EC] numbers) along bacterial and archaeal genomes and how this distribution has influenced the metabolic pathways in their current form. To this end, the information of 6,467 organisms with metabolic information that has been deposited in the KEGG database was assessed in terms of their enzymatic repertoires. This analysis provides clues about the functional constraints associated with the enzymatic repertoire of Bacteria and Archaea.

## Materials and methods

### Prokaryotic genomes

A total of 6145 bacterial and 322 archaeal complete genomes used in this study were downloaded from the NCBI genome database (18/03/22), using the ftp server (https://ftp.ncbi.nlm.nih.gov/genomes). These genomes corresponded to the list of organisms deposited in the KEGG database. KEGG annotations were obtained using the KEGG REST API (https://www.kegg.jp/kegg/rest/, October 2022). *Bacteria* (total numbers of genomes per division are shown in parentheses) included the following divisions (according to the NCBI classification system): *Acidobacteria* (15), *Actinobacteria* (907), *Alphaproteobacteria* (715), *Aquificae* (11), *Atribacterota* (1), *Bacteria incertae sedis* (21), *Bacteroidetes* (392), *Bathyarchaeota* (1), *Betaproteobacteria* (480), *Caldiserica* (1), *Calditrichaeota* (1), *Candidatus “*Thermoplasmatota” (10), *Chlamydiae* (123), *Chlorobi* (14), *Chloroflexi* (32), *Chrysiogenetes* (1), *Cyanobacteria* (101), *Deferribacteres* (5), *Deinococcus-Thermus* (25), *Deltaproteobacteria* (105), *Dictyoglomi* (2), *Elusimicrobia* (4), *Epsilonproteobacteria* (211), *Fibrobacteres* (2), *Firmicutes-Bacilli* (873), *Firmicutes-Clostridia* (248), *Firmicutes*-Others (54), *Fusobacteria* (28), *Gammaproteobacteria-Enterobacteria* (487), *Gammaproteobacteria*-Others (901), *Gemmatimonadetes* (4), *Nitrospinia* (1), *Nitrospirae* 31(10), Other *Fibrobacteres-Chlorobi-Bacteroidetes* or FCB group (3), Other *Chlamydiae/Verrucomicrobia* or PVC group (3), Other *Terrabacteria* group (3), Other proteobacteria (23), *Planctomycetes* (47), *Spirochaetes* (88), *Synergistetes* (5), *Tenericutes* (144), *Thermodesulfobacteria* (5), *Thermotogae* (28), *Verrucomicrobia* (19), unclassified *Bacteria* (1), and *Coprothermobacterota* (1). *Archaea* included the following divisions: *Crenarchaeota* (63), *Euryarchaeota* (215), *Lokiarchaeota* (1), *Micrarchaeota* (3), *Nanoarchaeota* (2), *Nanohaloarchaeota* (1), *Korarchaeota* (1), *Thaumarchaeota* (23), and unclassified *Archaea* (2). (See Table S1 for a complete description of all organisms considered in this analysis).

### Identification of EC enzyme classes

The Catalytic Families (CatFam version 2.0) program was used to scan the complete set of protein encoding genes associated with the 6,467 bacterial and archaeal genomes, using default conditions. CatFam generates sequence profiles to assign catalytic activities on protein sequences, minimizing the rate of false-positive predictions [11]. These annotations were merged with the enzymatic annotations from the KEGG database per genome^1^. In this regard, a protein can be associated with the same EC number, both CatFam and KEGG assignments; otherwise, if the enzyme is annotated with different EC numbers, both are considered.

### Genome and window size

To determine the association between total number of enzymes and genome sizes, the organisms were divided into windows of similar sizes, considering their number of Open Reading Frames (ORFs). We used Sturges’s formula, which groups different values in equal classes, as follows: *k* = 1 + log_2_*N*, where *k* is the number of equal classes and *N* the number of data, rounding to the nearest integer, the *k* value. Then, the width of classes was determined with the following equation: c = *R/k*, where R = high value (11,518 ORFs) – low value (116 ORFs) and corresponds to genome size. Values resulting from the application of the formulas were *k* = 13 and *c* = 877; thus, 13 windows without overlaps were used, with a width of 877 ORFs. The regression analysis was performed in R programming and RStudio version 4. 1.1 (http://www.rstudio.com).

### Statistical analyses

To determine the statistical significance of the seven EC classes proportions from each genome, an analysis of variance (ANOVA) followed by a Tukey Honestly-significant-difference (HSD) test was achieved, with a *p* value of <0.05. The boxplot and all statistical tests were done with R programming language, and RStudio version 4.1.1 (http://www.rstudio.com).

### Clustering analysis

The relative abundances of the seven EC classes were calculated as a function of the total enzymes per metabolic map deposited in the KEGG database. Therefore, the abundances are associated with a scale color, in minor proportion (0.0) and major (0.5) per metabolic map. Posteriorly, a hierarchical clustering approach using a Manhattan distance, and average linkage algorithm was done. This analysis was performed with R programming language and RStudio version 4. 1.1, with the packages readxl, pheatmap, ggplot2, colorspace, grid and RcolorBrewer (http://www.rstudio.com).

## Results and discussion

### The repertoire of enzymatic proteins in bacterial and archaeal genomes

**Fig. 1:**
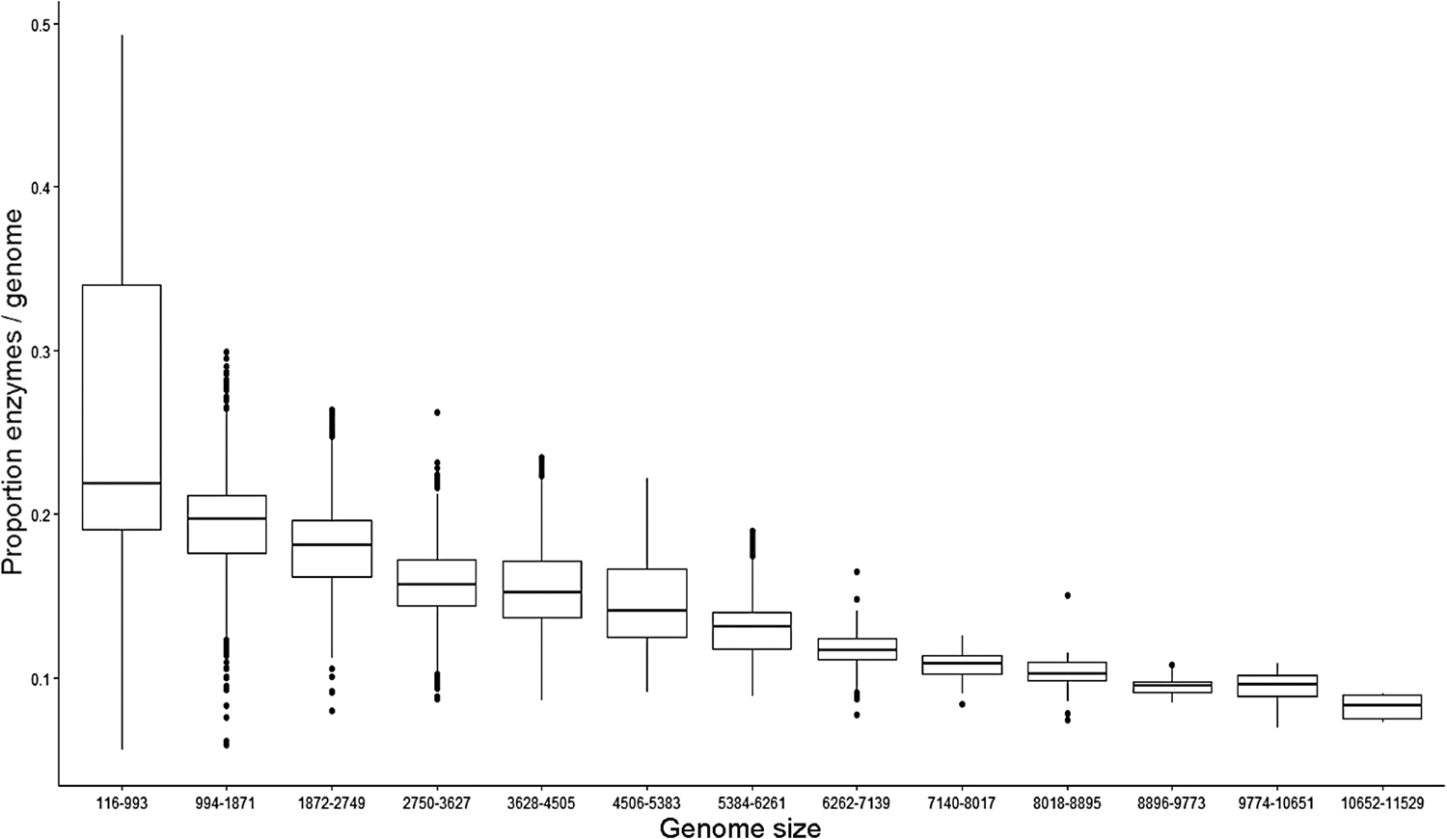
Proportion of enzymes in *Bacteria* and *Archaea* genomes. The number of enzymes was normalized against the total number of ORFs per genome. On the X-axis is the interval (genome size, in ORFs). On the Y-axis is the proportion of enzymes per genome. Each point represents a genome

A collection of 6,467 bacterial and archaeal genomes was scrutinized to determine the repertoire of enzymatic proteins. From this analysis, we identified that the distribution of the enzymes with respect to genome size (number of ORFs) followed a power-law behavior, with a correlation coefficient (*r*^2^) of 0.8424. The exponent of the power-law function was 0.7155 (Figure S1), which was within the range of exponents of protein families with functions related to metabolism and cell transport [12, 13]. This analysis showed that organisms with small genomes (ORFs) contain a small number of enzymes, in contrast to organisms with large genomes (ORFs), which contain a large number of enzymes (Figure S1). This finding is consistent with the notion of general scaling laws in the distribution of protein families, as already has been suggested [14–17]. To exclude the overrepresentation of sequenced bacterial genomes versus archaeal genomes or an uneven sampling of genomes with different size-ranges, we analyzed the abundance and distributions of enzymes in equivalent sets of Bacteria and Archaea*.* This involved randomly selecting a subset of 322 bacterial genomes (repeated 100 times), and comparing them with the repertoire of enzymes found in archaeal genomes Table S2*.* From this analysis, we identified that the distribution of enzymes is not influenced by the overrepresentation of bacterial genomes, i.e., they follow a similar trend like the general distribution show in Fig. 1.

To evaluate the proportion of enzymes by organism, we normalized the total enzymes as a function of the genome size (number of ORFs) (Fig. 1). From this normalization, we identified that the organisms with small genomes have a large proportion of enzymes (around 19% of their proteins are enzymes); for instance, 24.7% of the proteins identified in the small bacterium *Candidatus* “*Tremblaya phenacola*,” which is a symbiont of insects and is associated with rearrangement and loss of redundant genes [18], are enzymes. In contrast, organisms with the largest genome sizes (10,652 to 11,529 ORFs) exhibit a minor proportion of enzymes, on average 8% of enzymes (Fig. 1 and Table S3). For instance, in the cyanobacterium *Nostoc flagelliforme* (10825 ORFs), which under desiccation stress protects itself by induction of catalase, proteases, sucrose synthase, trehalose biosynthesis, and maltodextrin [19], only 7% of its proteins are enzymes.

### Abundance of enzyme EC classes in bacterial and archaeal genomes

**Fig. 2:**
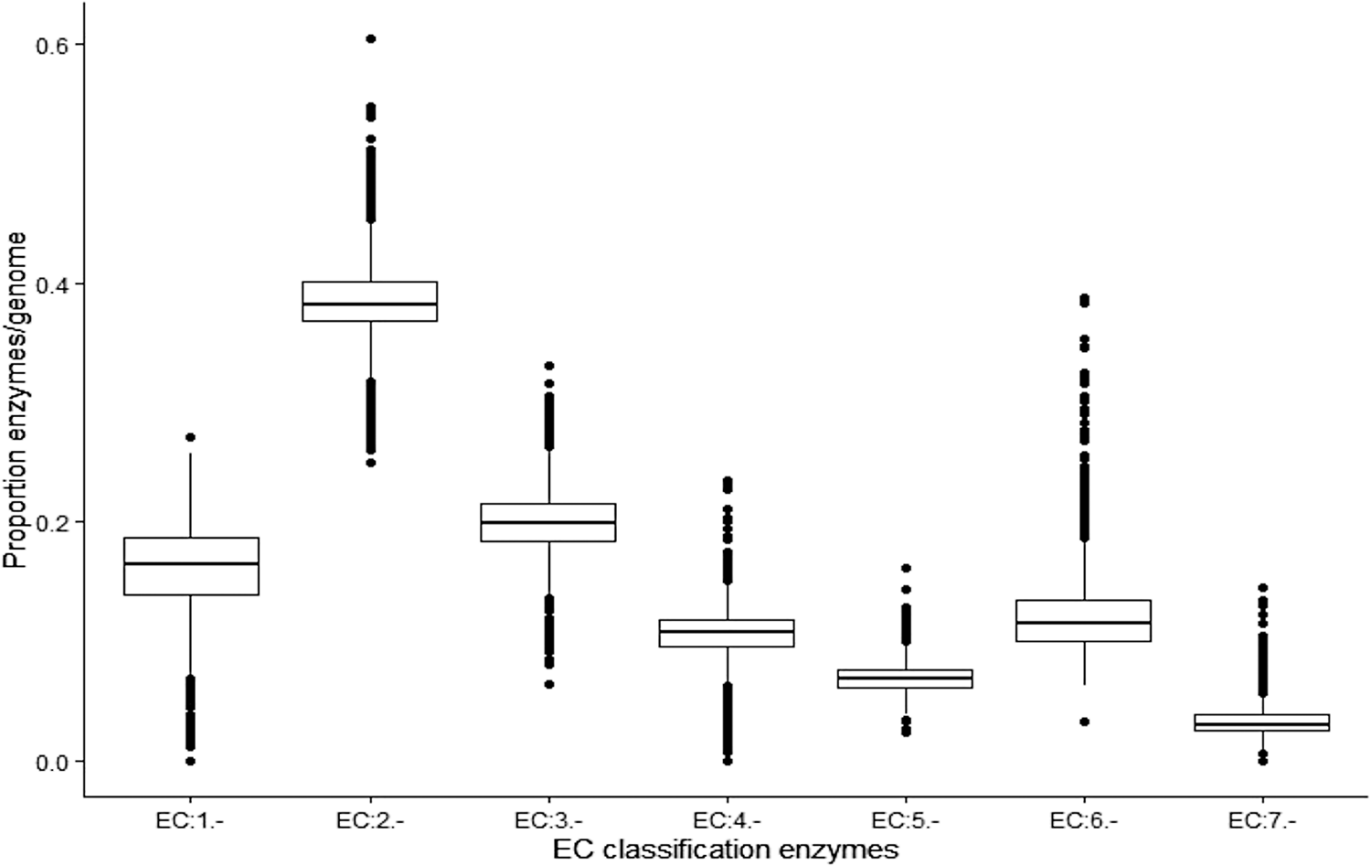
Proportion of EC classes in *Bacteria* and *Archaea* genomes. The abundances of the seven enzymatic classes (EC:1.- to EC:7.-) were normalized considering the number of ORFs per genome. Each point represents a genome. ANOVA and Tukey HSD test were performed, identifying significant differences (*p* < 2.2e^-16^) between all enzymatic classes

To characterize the enzymatic repertoire identified in the bacterial and archaeal genomes, we evaluated its distribution and abundance in terms of their functional classification, *i.e.,* considering the seven enzymatic classes. From this analysis, we found that transferases (EC:2.-) were the most abundant enzymes, followed by hydrolases and oxidoreductases. In minor proportions were found isomerases (EC:5.-), and translocases (EC:7-.). (Table 1 and Fig. 2). A similar result was found when Bacteria and Archaeal genomes were analyzed separately (Figure S2).

**Table 1:** Abundance of EC classes

EC number	EC classification	Abundance
EC:2.-	Transferases	36%
EC:3.-	Hydrolases	19%
EC:1.-	Oxidoreductases	16%
EC:6.-	Ligases	10%
EC:4.-	Lyases	10%
EC:5.-	Isomerases	6%
EC:7-.	Translocases	3%

Our results correlated with previous analyses that show that essential-genes identified in 14 bacterial genomes, considering the DEG database, are enriched with enzymatic functions [20] These “essential enzymes” are mainly associated with ligase activities (especially those forming carbon-oxygen bonds and carbon-nitrogen bonds), nucleotidyl transferases, and phosphotransferases [20]. In this regard, *Firmicutes* and *Deltaproteobacteria* are known to present sulfur-reducing metabolism; sulfur is an essential element for life, and the metabolism of organic sulfur compounds plays an important role in the global sulfur cycle, which is common in extremophile microorganisms [21]. Organisms of these phyla participate in the nitrogen and sulfur cycle metabolism associated with energy production, which would explain the large proportions of transferases (EC:2.-) and oxidoreductases (EC1.-) necessary for the survival of microorganisms in extreme and adverse environments [15, 21, 22]. In this regard, sulfur metabolism, at least in the presence of oxygen, requires a significant consumption of energy as well as the maintenance of a very low oxido-reduction potential. In *E. coli*, 45% of the enzymes associated with this sulfur metabolism belong to the class EC:2-, followed by the class EC:1.-, with 35%. For instance, EC: 2.7.-, such as the subunits of the ATP, sulfate adenylyltransferase, and adenylylsulfate kinase (CysN, CysD and CysS), or those proteins belonging to the flavoprotein and hemoprotein subunits (CysJ and CysI, with EC:1.8) [23].

Therefore, this distribution suggests that enzyme-catalyzed transfer and oxidoreduction reactions are highly abundant in metabolism, probably because metabolic processes can be seen as the movement of electrons between molecules, often capturing some of the energy released as the electrons move from high-energy to lower-energy states [24].

Another example corresponds to the phyla *Betaproteobacteria* and *Gammaproteobacteria* and *Thaumarchaeota*, which carry out an oxidation reaction to transform ammonium to nitrite. Nitrification includes a second oxidation step of converting nitrite to nitrate, as described for *Nitrobacter* and *Nitrospira* bacteria [25, 26]. The key enzymes involved are described as ammonia monooxygenase, hydroxylamine dehydrogenase, nitric oxide oxidase, and nitrite oxidoreductase, which are involved in the metabolism of nitrogen. Whereas a high number of enzymes classified as oxidoreductases and translocases have been associated with extreme environments, an overrepresentation of these enzymatic activities for a free lifestyle promotes the conservation and reduction in the genome size for useful maintenance genes, which correlates with our findings [25–27].

In the case of hydrolases (EC:3.-), (Fig. 2) which are associated with the intricate arrangement of the cell wall, especially peptidoglycan, lipoteichoic acids, or polyglutamate, these compounds during growth should have a constant and strict balance between degradation and biosynthesis, and hydrolases play a central role in bacterial cell wall remodeling [28].

The enzymes with ligase activities (EC:6.-), which are present in *Bacteria* and *Archaea*, are involved in central processes such as DNA replication, recombination, and repair [29], were found in small proportions in the enzymatic repertoire.

The lyases (EC:4.-) are capable of performing decarboxylation reactions involved in metabolic pathways such as photosynthesis, in which sunlight provides energy to drive carbon fixation; this reaction is a critical reaction in metabolic pathways like respiration and the tricarboxylic acid cycle [30], explaining the fact that a small proportion of reactions were identified in the dataset Fig. 2.

The isomerases (EC:5.-) are involved in catalyzing up to 4% of the biochemical reactions present in central metabolism, in particular, carbohydrate metabolism with functions like racemases, epimerases, and *cis-trans* isomerases [31]. Finally, translocases (EC:7-.) that have been involved in molecules moving across cell membrane, such as ions, represent 3% of the dataset [32].

Therefore, our results reinforce the notion that the enzymes are central for survival and reproduction, as observed in the enrichment of ligases and transferases in the essential genes, described in bacterial genomes [20]. In this regard, in network simulations, transferase activities were found to be associated with new metabolic pathways, in particular, with multifunctional enzymes as a consequence of dependence on the donor or acceptor metabolite [33, 34].

In addition, the enzyme classification system used in this work is based on the biochemical activities performed by each enzyme and groups them in terms of reaction similarity [35, 36], and does not consider the evolutionary relationships among the members. In this regard, a recent work of paralogos in the seven enzymatic classes, identified a high ratio of duplications in oxidoreductases, isomerases and translocases [15], identified in this work as the less abundant; as a function of the environmental adaptation. Therefore, intracellular organisms have a lesser ratio of duplicated enzymes, whereas free-living organisms show the highest ratios.

In summary, we consider that the abundances of EC numbers could allow innovation at the metabolic level, since they include multiple duplication events, allowing organisms to adapt to environmental changes [31], as already has been previously suggested [15]

### Enzymatic classes are associated with metabolic maps

**Fig. 3:**
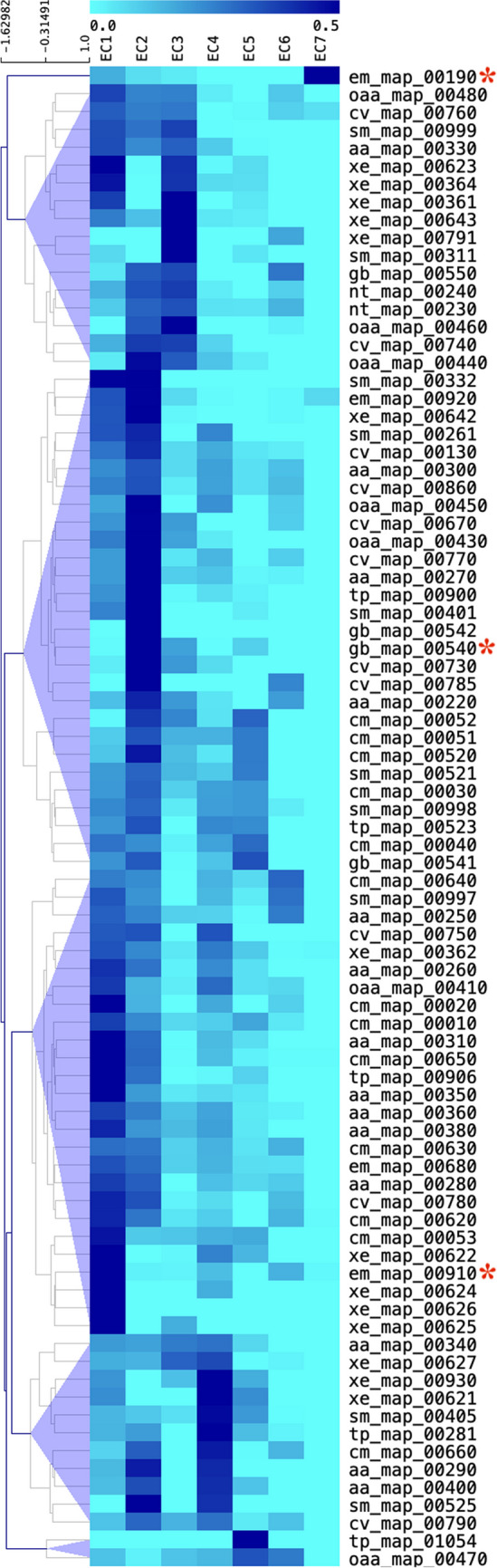
Distribution of EC enzyme class proportions among metabolic pathways. Each column denotes the enzyme EC class, and the rows denote each of the 84 metabolic pathways. Abbreviations related to metabolic processes: xe, xenobiotics; cv, cofactors and vitamins; cm, carbohydrates; aa, amino acids; sm, secondary metabolites; tp, terpenoids; oaa, oxaloacetic acid; gb, glycan biosynthesis; em, energy metabolism. The heatmap bar at the top of the figure indicates the relative abundances of EC enzyme classes, which are associated with scale color in minor proportion (0.0) and major (0.5). Four groups of maps were identified based on a hierarchical clustering approach by using a Manhattan distance supported with an average linkage algorithm, and the numbers in the upper left section show the weight scores, ranging from 0.6 – 1.0

To determine whether the identified enzymes exhibit a homogeneous distribution in all metabolic maps or are preferentially associated with a particular map, we evaluated their abundances and distributions based on the classification of EC numbers. To this end, a hierarchical clustering using a Manhattan distance supported with an average linkage algorithm was achieved, identifying four main clusters with scores of ≥0.6 (Fig. 3).

In the first cluster are included the maps of biosynthesis of other secondary metabolites (sm), as 00999 and 00311; xenobiotic biodegradation and metabolism (xe), 00623, 00361, 00364, 00643, 00364, and 00791; and cofactors and vitamin metabolism (cv), 00740 and 00760. In these maps, a high abundance of hydrolases (EC: 3.-), oxidoreductases (EC: 1.-), and transferases (EC: 2.-), were found.

In the second cluster, the maps associated with secondary metabolites (sm) 00261, 00332, 00401, 00521, and 00998 were identified. In these maps, we observed an abundance of oxidoreductases (EC: 1.-), transferases (EC: 2.-), lyases (EC: 4.-), and isomerases (EC: 5.-). In addition, we found maps associated with cofactor and vitamin metabolism (cv), 00130, 00860, 00670, 00770, 00730, 00130, and 00785; amino acid metabolism (aa) maps 00300, 00270, and 00220; with a high proportion of oxidoreductases (EC: 1.-), and transferases (EC: 2.-) (Fig. 3).

In the third cluster, the maps associated with carbohydrate metabolism (cm), 00640, 00020, 00010, 00650, 00630, 00620, and 00053; sulfur metabolism (sm) 0099; amino acid metabolism (aa) 00250, 00260, 00380, 00350, 00360, 00280, and 00380; and the metabolism of cofactors and vitamins (cv) (00750 and 00780) were identified. In general, these maps had a high proportion of oxidoreductases (EC: 1.-) and ligases (EC: 6.-) (Fig. 3).

In the fourth cluster, we found maps associated with xenobiotic metabolism (xe), such as 00627, 00930 and 00621; sulfur metabolism (sm), 00405; terpenoid metabolism (tp), 00281 and 01054; and carbohydrate metabolism (cm), 00660; with a high proportion of lyases (EC: 4.-) (Fig. 3).

Finally, one cluster of two elements, metabolism of terpenoids and polyketides (tp) 01054 and metabolism of other amino acids (oaa) 00470; and one orphan cluster, with one element (Oxidative phosphorylation, em, 00190), where a high proportion of ligases and translocases, were identified, respectively.

In summary, our results showed the relation of the enzymatic repertoires with maps focused on carbohydrate metabolism, as carbohydrates are the principal source of energy in bacteria [37]. In this regard, the Embden-Meyerhof pathway (EM) is the principal metabolism pathway to glucose degradation [38]. In *Archaea*, the genes that encode similar enzymes are also conserved for this pathway, for example, *Thermoproteus tenax* ferments ^13^C-glucose to low amounts of acetate and alanine via simultaneous operation of the EM pathway [39].

Therefore, the distribution of enzyme classes identified groups of maps with a high abundance of specific catalytic activities, such as the EC:3.- in the xenobiotic maps or the EC:2.- in amino acids and carbohydrates or the EC:7.- devoted to energy metabolism, among others, suggesting that metabolic maps have an overrepresentation of specific enzymatic activities.

### Nitrogen fixation and LPS biosynthesis functions contain a high proportion of specific EC classes

Microorganisms can live in an adverse environment because they are capable of adapting to it, which is achieved by mediating cellular metabolism through a great variety of biochemical reactions performed by enzymes. In this regard, diverse metabolic maps were found to be specifically associated with an EC class, such as nitrogen fixation and lipopolysaccharide (LPS) biosynthesis.

**Fig. 4:**
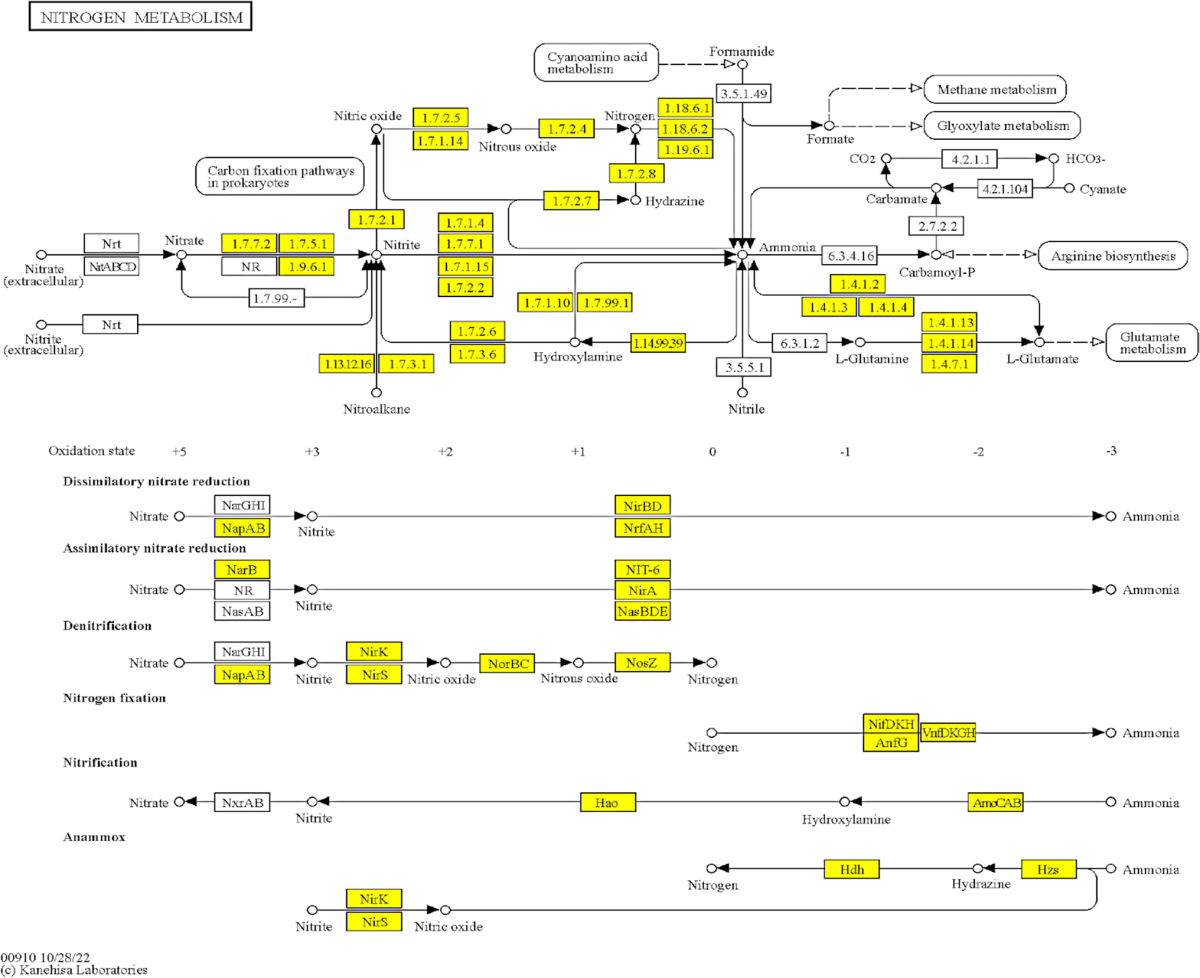
Nitrogen fixation metabolism (KEGG ID map00910) showing a high proportion of oxidoreductases (EC:1.-), indicated in yellow color. Map taken from https://www.genome.jp/dbget-bin/www_bget?map00910

In this regard, the nitrogen fixation pathway (map 00910) contains a high content of oxidoreductases (EC:1.-). The nitrogen cycle is based on the nitrogen oxidation state as nitrate (+5) or ammonia (-3) (Fig. 4). The map includes the seven canonical N-cycling pathways—nitrification, dissimilatory nitrate reduction, denitrification, dissimilatory nitrite reduction, assimilatory nitrate reduction, assimilatory nitrite reduction, and nitrogen fixation. In this pathway, 65% of the reactions are associated to the EC:1.- class, such as the denitrification process that consists of metabolizing the nitrite to nitric oxide, transforming by EC:1.7.2.5, EC:1.7.1.14 to nitrous oxide, and finally to nitrogen with the participation of EC:1.7.2.4. (Fig. 4).

**Fig. 5:**
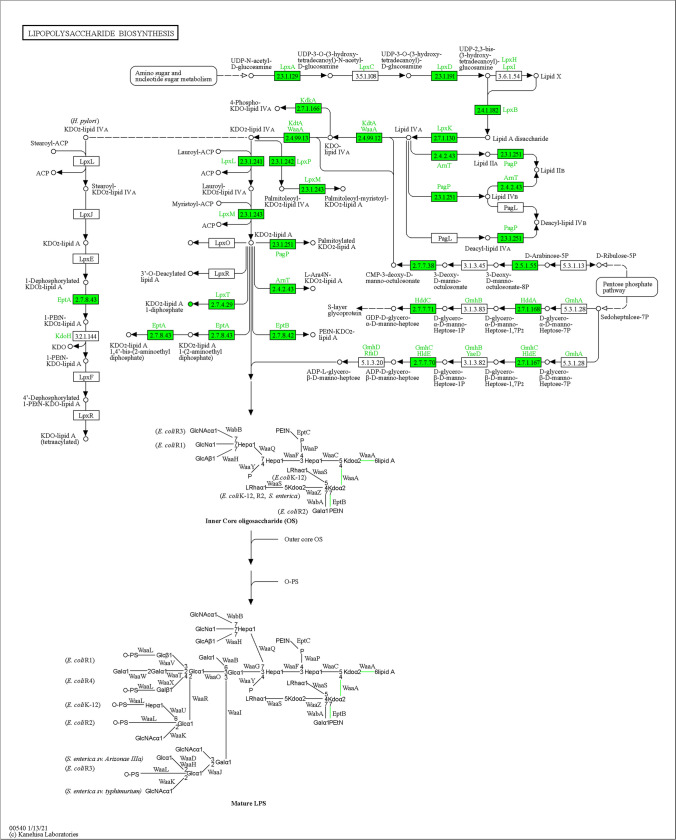
Biosynthesis of lipopolysaccharide (KEGG ID map00540) showing a high proportion of transferases (EC:2.-), indicated in green color. Map taken from https://www.genome.jp/dbget-bin/www_bget?map00540

The second example selected was Lipopolysaccharide (LPS) biosynthesis (map 00540), where 71% of the reactions correspond to transferases (EC:2-) (Fig. 5). UDP-*N*-acetylglucosamine acyltransferase (EC:2.3.1.129) catalyzes the first step in lipid A biosynthesis [40]. It transforms UDP-*N*-acetyl-d-glucosamine to UDP-3-O-(3 hydroxy tetradecanoic)-*N*-acetyl-d-glucosamine. This is metabolized to lipid A disaccharide by enzymes EC:2.4.1.182 and EC:2.7.1.130 to convert this to lipid IV_A_, followed by the conversion in KDO lipid IV_A_ for EC:2.4.9.12 and 2.4.9.143, and finally KDO lipid IV_A_ is condensed with inner core oligosaccharide by EC:2.3.251, EC:2.4.2.43, EC:2.7.8.42, and EC:2.7.4.2. In this regard, this functional conservation implies that the genes for each pathway associated with the LPS have a single origin, and are usually organized in clusters into the genome [40]. This organization probably makes it more likely that they will be transferred as a set of genes, thereby providing selection for this functional arrangement into the pathway.

**Fig. 6:**
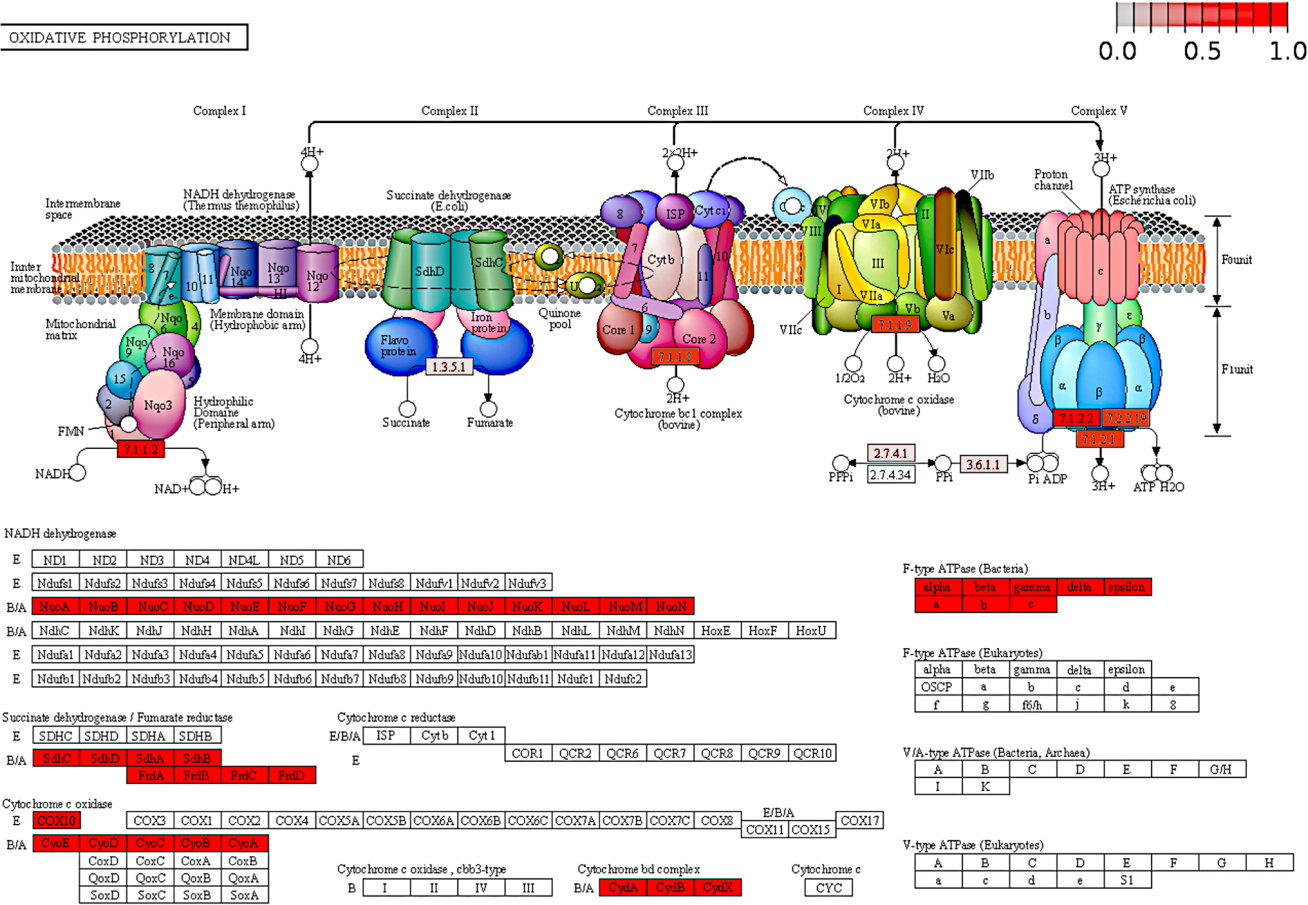
Oxidative phosphorylation metabolism (KEGG ID map00190). The translocases (EC:7.), indicated in red color, are involved in the process of transporter ions, and produce energy during the process. Map taken from https://www.genome.jp/dbget-bin/www_bget?map00190

Finally, translocases (EC:7.-) are mainly associated with the oxidative phosphorylation process metabolism (map 00190). In this map, NADH-quinone oxidoreductase (EC:7.1.1.2), cytochrome ubiquinol oxidase unit I (EC: 7.1.1.7), and V/a-type H^+^/Na^+^ transporting ATPase subunit A (EC:7.1.2.2) stand out; all these enzymes are involved in the H^+^ translocation through the membrane associated with the electron transport chain (Fig. 6) [36]. Interestingly, we found enzymes of the class EC:7.- highly abundant in *Thaumarchaeota* despite that it being an *Archaea* that lives in extreme environments, whereas in *Bacteria*, we found abundant translocases in *Alphaproteobacteria* and *Betaproteobacteria*; in fact, these groups (*Thaumarchaeota* and *Proteobacteria*) include organisms with small genomes [22, 41]. Thus, our findings suggest a correspondence between catalytic activities and metabolic processes, mainly involved in energy production, detoxification, capture of nutrients, antibiotic metabolism, and degradation of aromatic hydrocarbon elements used by microorganisms as survival strategies.

## Conclusions

In this work, we evaluated 6,467 genomes of *Bacteria* and *Archaea*, corresponding to 29 different phyla. Our approach analyze the enzymatic repertoire encoded in the genomes of these domains of life. To this end, the first level of the EC number was considered because we tried to catalog the functional repertoire of these proteins and their recruitment into the evolution of metabolic pathways as a broad metabolic function. Therefore, the first level of classification gives us the general type of reaction for this purpose; although we understand that the EC numbers are associated with diverse functions, protein families, and different folds. From this analysis, we determined that the enzymes follow a power-law distribution in relation to the genome size. In addition, we found that the smallest genomes had the major proportion of enzymes and even conserved the major presence of oxidoreductases and transferases; whereas, larger genomes presented the minor proportion of enzymes but showed the major proportion of translocases. Therefore, we evaluated the total proportion of EC enzyme classes with different metabolic maps, and we observed that the proportion of enzymes was conserved in the genomes presented, in descending-order proportion: transferases (EC:2.-), hydrolases (EC:3.-), oxidoreductases (EC:1.-), ligases (EC:6.-), lyases (EC:4.-), isomerases (EC:5.-), and translocases (EC:7-.). Interestingly, we determined that most of metabolism pathways for xenobiotics, cofactors and vitamins, carbohydrates, amino acids, glycans, and energy are conserved in terms of the catalytic activities, among the metabolic maps analyzed.

## Supplementary information

ESM 1 **Figure S1. Proportion enzymes per genome size.** Scatterplot shows the relationship between total enzymes and genomes size (ORFs) (A), *Bacteria* (B) and *Archaea* (C) with the behavior of the power-law function. The equation for adjustment and the *R*^2^ value are shown; note that the *R*^2^ value is higher and positive following the scaling law of the number of enzymes and the genome size. (PNG 70 kb)

High resolution image (TIFF 8.86 MB)

(PNG 68 kb)

High resolution image (TIFF 11.1 MB)

(PNG 42 kb)

High resolution image (TIFF 11.1 MB)

ESM 2 Figure S2. **Proportion of EC classes in** ***Bacteria*** **(white) and** ***Archaea*** **(gray) genomes.** The abundances of the seven enzymatic classes (EC:1.- to EC:7.-) were normalized considering the number of ORFs per genome. Each point represents a genome. (PNG 25 kb)

High resolution image (TIFF 8.05 MB)

ESM 3 **Table S1. Complete description of all organisms considered in the analysis.** Characteristics analyzed in the study included KEGG_ID, KEGG_name taxonomy, phylum, Genome ID, Genome_name, number of ORFs, total of enzymes, total of enzymes by EC class (EC:1.-, EC:2.-, EC:3.-, EC:4.-, EC:5.-, EC:6.-, EC:7.-), total enzyme proportions, and enzyme proportions by EC class. (XLSX 2043 kb)

ESM 4 Table S2. Abundance and distributions of enzymes in equivalent genome datasets of Bacteria and Archaea. (DOCX 13 kb)

ESM 5 **Table S3. Enzymatic repertoire analyses related to prokaryotic genome.** Description of genomic information organization showed the genome size intervals, the phylum that corresponds to each interval, average numbers of ORFs and enzymes, and total numbers and relative percentages of EC enzyme classes, with a microorganism example corresponding to each genome size interval analyzed. (DOCX 18 kb)
