# Supplementary figures and images for: Exploring the enzymatic repertoires of Bacteria and Archaea and their associations with metabolic maps
Source: Braz J Microbiol. 2024 Jul 25;55(4):3147–57. doi: 10.1007/s42770-024-01462-3 (PMC11711735; doi:10.1007/s42770-024-01462-3)

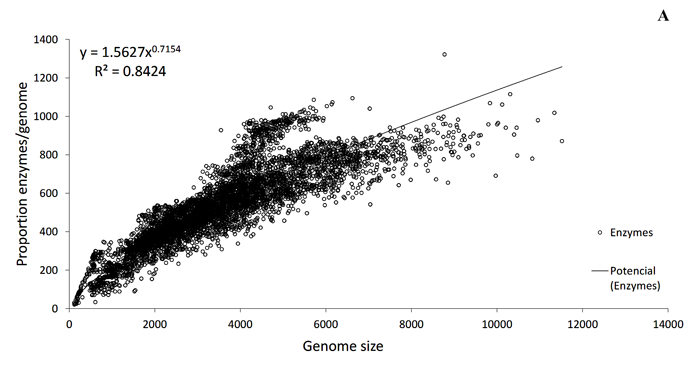

Supplement: Supplementary file 1 — Figure S1. Proportion enzymes per genome size. Scatterplot shows the relationship between total enzymes and genomes size (ORFs) (A), Bacteria (B) and Archaea (C) with the behavior of the power-law function. The equation for adjustment and the R2 value are shown; note that the R2 value is higher and positive following the scaling law of the number of enzymes and the genome size. (PNG 70 kb) [file 42770_2024_1462_Fig7_ESM.png]

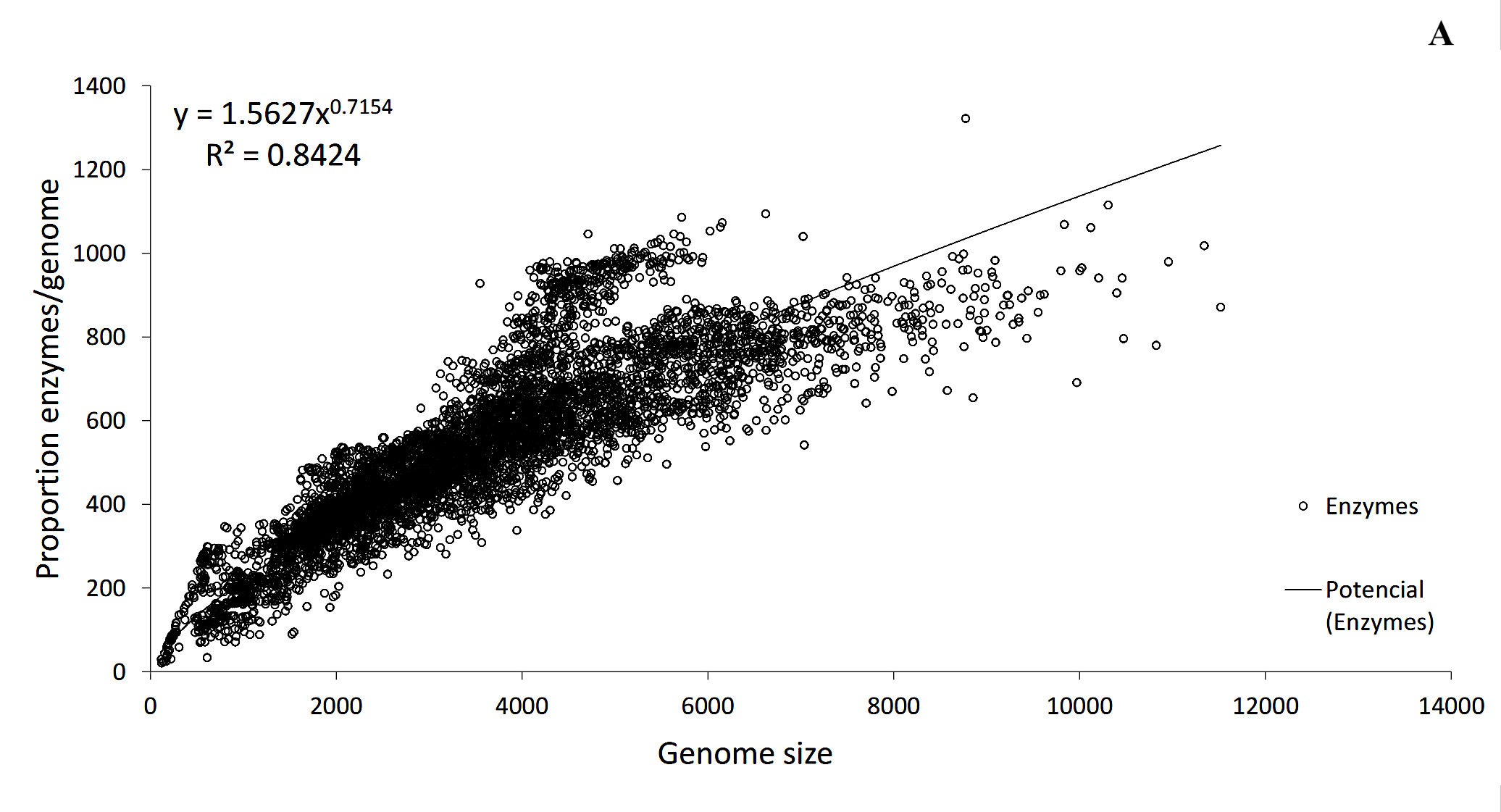

Supplement: Supplementary file 2 — High resolution image (TIFF 8.86 MB) [file 42770_2024_1462_MOESM1_ESM.tiff]

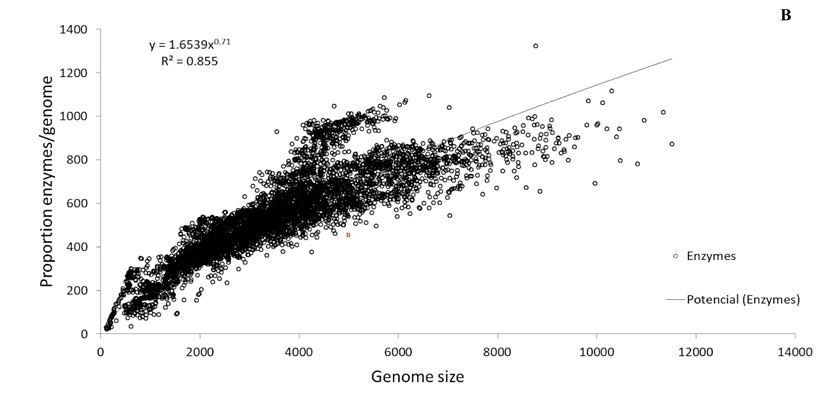

Supplement: Supplementary file 3 — (PNG 68 kb) [file 42770_2024_1462_Fig8_ESM.png]

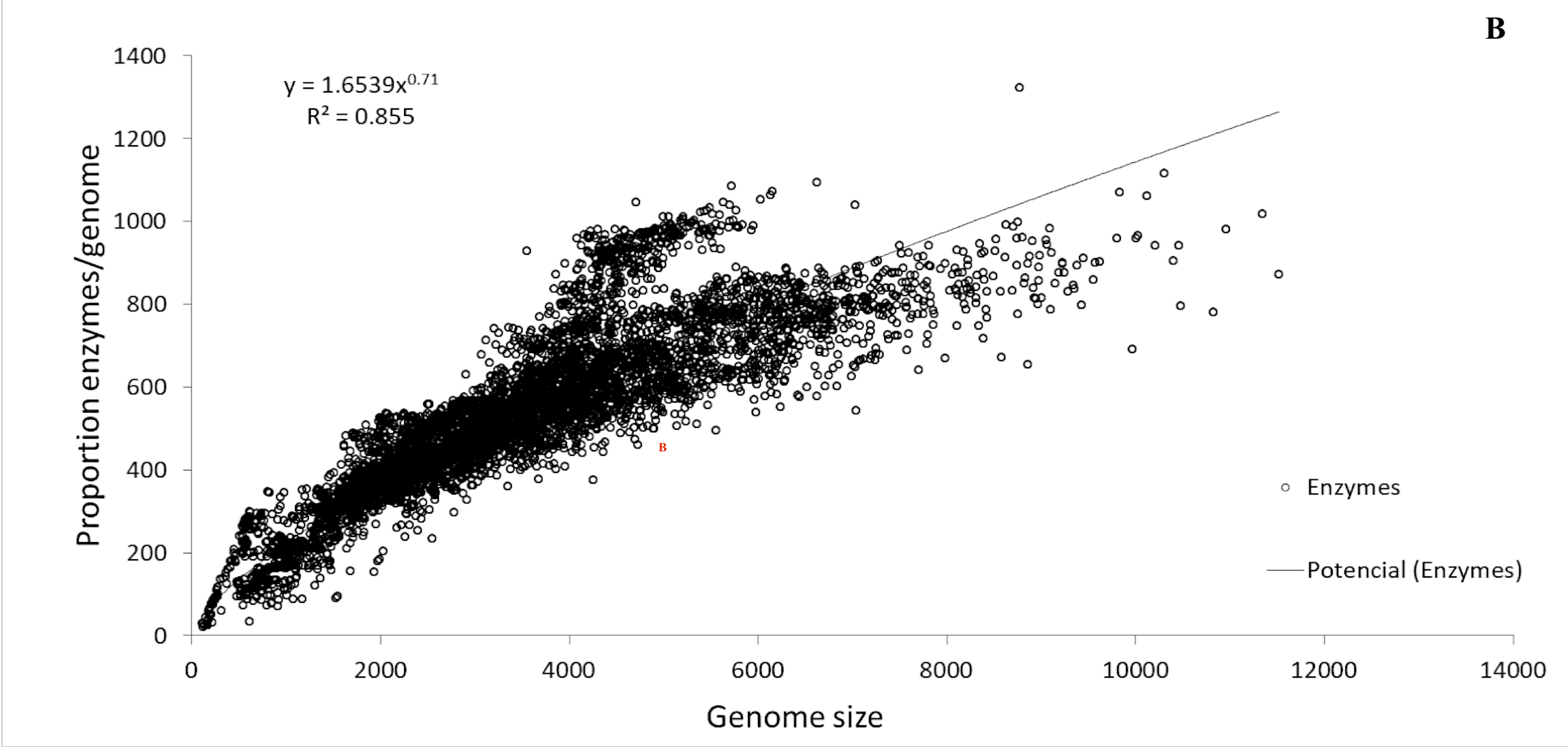

Supplement: Supplementary file 4 — High resolution image (TIFF 11.1 MB) [file 42770_2024_1462_MOESM2_ESM.tiff]

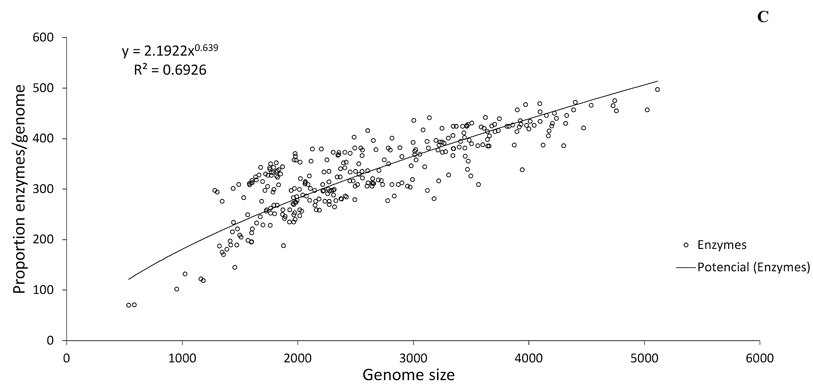

Supplement: Supplementary file 5 — (PNG 42 kb) [file 42770_2024_1462_Fig9_ESM.png]

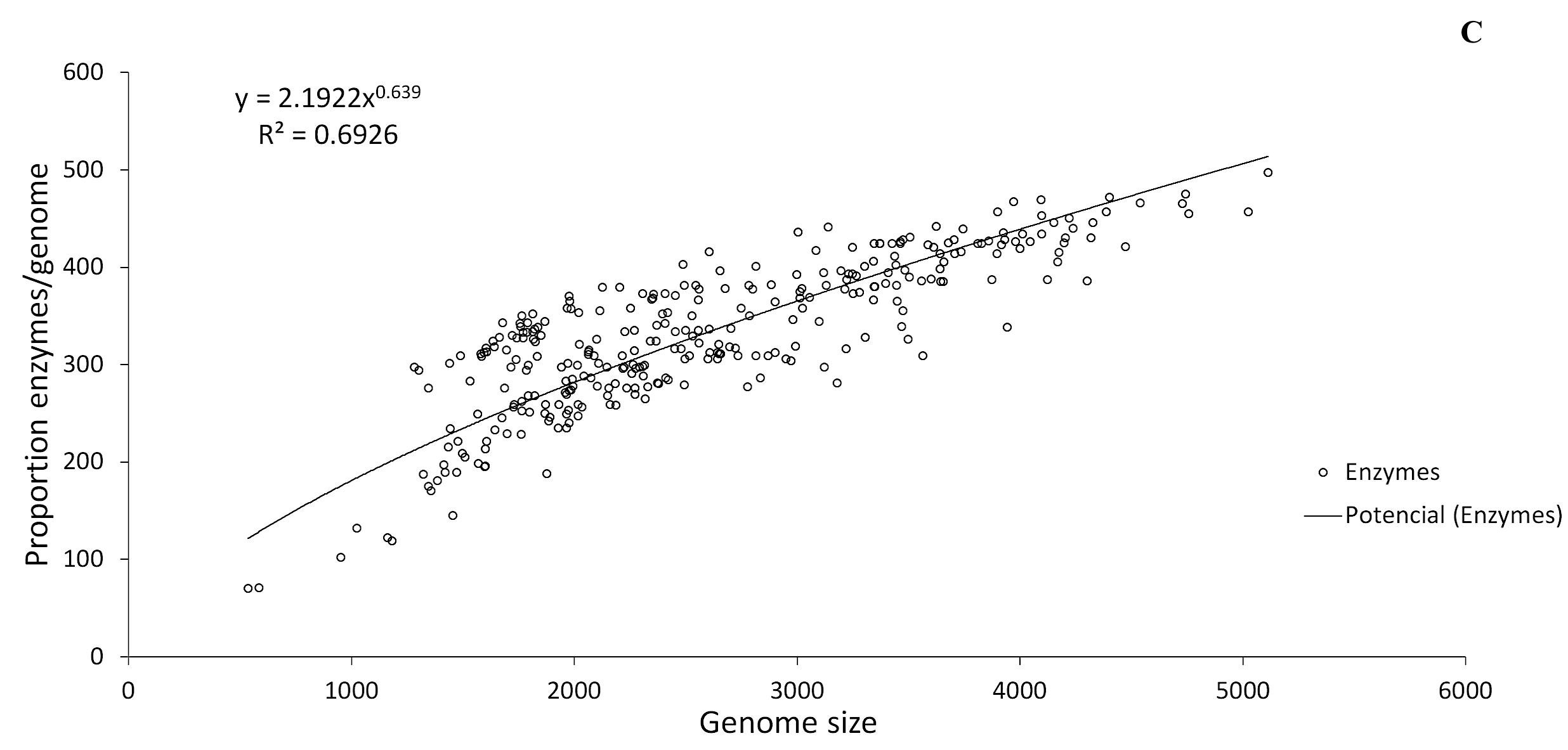

Supplement: Supplementary file 6 — High resolution image (TIFF 11.1 MB) [file 42770_2024_1462_MOESM3_ESM.tiff]

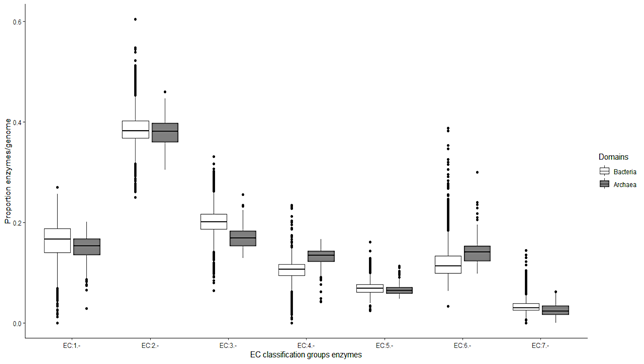

Supplement: Supplementary file 7 — Figure S2. Proportion of EC classes in Bacteria (white) and Archaea (gray) genomes. The abundances of the seven enzymatic classes (EC:1.- to EC:7.-) were normalized considering the number of ORFs per genome. Each point represents a genome. (PNG 25 kb) [file 42770_2024_1462_Fig10_ESM.png]

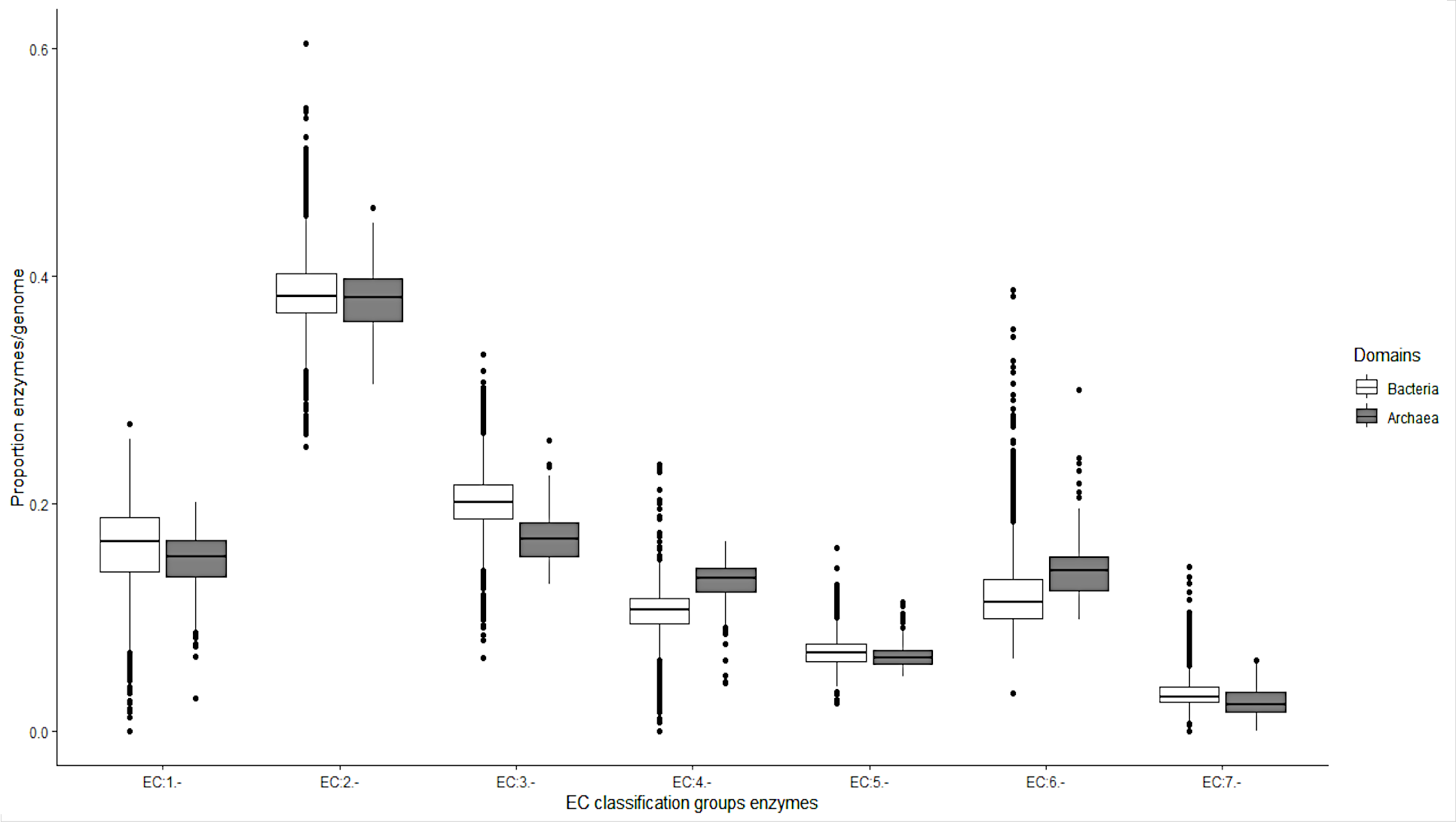

Supplement: Supplementary file 8 — High resolution image (TIFF 8.05 MB) [file 42770_2024_1462_MOESM4_ESM.tiff]
