# Supplementary material for: Exploring the enzymatic repertoires of Bacteria and Archaea and their associations with metabolic maps
Source: Braz J Microbiol. 2024 Jul 25;55(4):3147–57. doi: 10.1007/s42770-024-01462-3 (PMC11711735; doi:10.1007/s42770-024-01462-3)
Supplement: Supplementary file 10 — Table S2. Abundance and distributions of enzymes in equivalent genome datasets of Bacteria and Archaea. (DOCX 13 kb) [file 42770_2024_1462_MOESM6_ESM.docx]

Table S2. Abundance and distributions of enzymes in equivalent genome datasets of Bacteria and Archaea.

|  | **Bacteria** | **Bacteria subset** | **Archaea** |
| --- | --- | --- | --- |
| Total of genomes | 6,146 | 322 | 322 |
| Average ORFs | 3,501 | 3,499 +-86 | 2,566 |
| Average of enzymes | 533 | 532 +-10 | 328 |
| r2 | 0.86 | 0.86 +- 0.01 | 0.8392 |
