# Supplementary material for: Exploring the enzymatic repertoires of Bacteria and Archaea and their associations with metabolic maps
Source: Braz J Microbiol. 2024 Jul 25;55(4):3147–57. doi: 10.1007/s42770-024-01462-3 (PMC11711735; doi:10.1007/s42770-024-01462-3)
Supplement: Supplementary file 11 — Table S3. Enzymatic repertoire analyses related to prokaryotic genome. Description of genomic information organization showed the genome size intervals, the phylum that corresponds to each interval, average numbers of ORFs and enzymes, and total numbers and relative percentages of EC enzyme classes, with a microorganism example corresponding to each genome size interval analyzed. (DOCX 18 kb) [file 42770_2024_1462_MOESM7_ESM.docx]

**Table S2. Enzymatic repertoire analyses related to prokaryotic genome signatures.** Description of genomic information organization showed the genome size intervals, the phylum that corresponds to each interval, average numbers of ORFs and enzymes, and total numbers and relative percentages of EC enzyme classes, with a microorganism example corresponding to each genome size interval analyzed.

| Phylum | Interval  genome size  (ORFs) | Average  ORFs | Average  Enzymes  (%) | EC classes (%) | | | | | | | Organism | Comment |
| --- | --- | --- | --- | --- | --- | --- | --- | --- | --- | --- | --- | --- |
|  |  |  |  | 1 | 2 | 3 | 4 | 5 | 6 | 7 |  |  |
| Gammaproteobacteria - Others  Gammaproteobacteria - Enterobacteria  Bacteroidetes  Tenericutes  unclassified Bacteria  Alphaproteobacteria  Betaproteobacteria  Elusimicrobia  Spirochaetes  Bacteria incertae sedis  Chlamydiae | 116 – 993 | 175 | 25 | 13 | 37 | 20 | 8 | 8 | 18 | 4 | *Candidatus Tremblaya phenacola* | This organism is described as P-endosymbiont of insects that contain obligate mutualism bacteria, which is essential for insect survival.  The microenvironment is associated with massive rearrangements and loss of redundant genes. |
| Chlamydiae  Tenericutes  Alphaproteobacteria  Spirochaetes  unclassified Archaea  Bacteria incertae sedis  Micrarchaeota  Firmicutes - Bacilli  Gammaproteobacteria - Others  Actinobacteria  Deltaproteobacteria  Nanohaloarchaeota  Firmicutes - Others  Euryarchaeota  Fusobactera  Cyanobacteria  Crenarchaeota  Epsilonproteobacteria  Bacteroidetes  Elusimicrobia  Firmicutes - Clostridia  Chloroflexi  Coprothermobacterota  Synergistetes  Thaumarchaeota  Betaproteobacteria  Aquificae  Candidatus Thermoplasmatota  Deinococcus-Thermus  Caldiserica  Thermodesulfobacteria  Korarchaeota  Thermotogae  Dictyoglomi  Bathyarchaeota  Gammaproteobacteria - Enterobacteria  Other FCB group  Other PVC group | 994-1871 | 1556 | 19 | 14 | 39 | 19 | 10 | 8 | 15 | 3 | *Bifidobacterium animalis* subsp. *lactis* ATCC 27673 | This microorganism is considered health-promoting probiotic microorganisms and used in the formulation of foods.  The environment is totally controlled reduced the variability. |
| Betaproteobacteria  Euryarchaeota  Firmicutes - Bacilli  Gammaproteobacteria - Others  Thermotogae  Actinobacteria  Epsilonproteobacteria  Synergistetes  Crenarchaeota  Alphaproteobacteria  Thaumarchaeota  Aquificae  Cyanobacteria  Dictyoglomi  Bacteroidetes  Firmicutes - Others  Fusobacteria  Tenericutes  Firmicutes - Clostridia  Candidatus Thermoplasmatota  Chlamydiae  Chlorobi  Acidobacteria  Nitrospirae  Deferribacteres  Verrucomicrobia  Other proteobacteria  Chloroflexi  Gammaproteobacteria - Enterobacteria  Thermodesulfobacteria  Spirochaetes  Deinococcus-Thermus  Planctomycetes  Deltaproteobacteria  Other PVC group  Other Terrabacteria group  Chrysiogenetes | 1872-2749 | 2311 | 18 | 15 | 39 | 19 | 11 | 8 | 13 | 3 | *Bacteroidales* strain CF | This organism has a genome size of ~2.66 Mb, with an average G+C content of 42%, It has been described as microorganism able to dechlorinates >50 mg/liter chloroform or 1,1,1-trichloroethane (methyl chloroform), in addition and this bacterium is able to ferment l-lactate and ethanol. |
| Actinobacteria  Firmicutes - Bacilli  Bacteroidetes  Betaproteobacteria  Alphaproteobacteria  Gammaproteobacteria - Others  Firmicutes - Clostridia  Atribacterota  Cyanobacteria  Verrucomicrobia  Deltaproteobacteria  Spirochaetes  Crenarchaeota  Firmicutes - Others  Euryarchaeota  Thermotogae  Other Terrabacteria group  Other proteobacteria  Epsilonproteobacteria  Fibrobacteres  Fusobacteria  Deinococcus-Thermus  Gammaproteobacteria - Enterobacteria  Acidobacteria  Planctomycetes  Chloroflexi  Deferribacteres  Bacteria incertae sedis  Synergistetes  Thaumarchaeota  Other FCB group  Nitrospirae  Gemmatimonadetes  Nitrospinia | 2750-3627 | 3221 | 15 | 16 | 39 | 20 | 11 | 7 | 12 | 3 | *Desulfovibrio vulgaris* RCH1 | It is a sulfate-reducing bacteria member able to use sulfate as an electron acceptor in anaerobic respiration. This bacterium has and those bacteria have been associated as important described in the process of bioremediation of toxic metals and radionuclides such as chromium and uranium. |
| Alphaproteobacteria  Firmicutes - Clostridia  Gammaproteobacteria - Others  Bacteroidetes  Firmicutes - Bacilli  Cyanobacteria  Gammaproteobacteria - Enterobacteria  Betaproteobacteria  Deinococcus-Thermus  Euryarchaeota  Actinobacteria  Spirochaetes  Planctomycetes  Deltaproteobacteria  Fusobacteria  Acidobacteria  Other proteobacteria  Chloroflexi  Verrucomicrobia  Firmicutes - Others  Gemmatimonadetes  Lokiarchaeota  Nitrospirae  Other PVC group  Calditrichaeota | 3628-4505 | 4107 | 15 | 18 | 38 | 21 | 11 | 7 | 10 | 3 | *Luteolibacter ambystomatis* | It belongs to the family *Verrucomicrobia* phylum *Verrucomicrobia* involved in nitrate reduction activity-dependent species. |
| Actinobacteria  Firmicutes - Bacilli  Cyanobacteria  Gammaproteobacteria - Enterobacteria  Gammaproteobacteria - Others  Alphaproteobacteria  Firmicutes - Clostridia  Chloroflexi  Bacteroidetes  Euryarchaeota  Acidobacteria  Betaproteobacteria  Planctomycetes  Verrucomicrobia  Deltaproteobacteria  Firmicutes - Others  Nitrospirae  Other Terrabacteria group | 4506-5383 | 4962 | 14 | 18 | 38 | 21 | 11 | 6 | 9 | 3 | *Paenibacillus polymyxa* CR1 | It is a plant-growth promoting rhizobacteria involved in antimicrobial-activity, nitrogen fixation, phosphate solubilization, plant hormone production and/or lignocellulose degradation. It is involved in treatment of chemical fertilizer and pesticides for environmental friendlier. |
| Actinobacteria  Gammaproteobacteria - Others  Bacteroidetes  Firmicutes - Bacilli  Alphaproteobacteria  Gammaproteobacteria - Enterobacteria  Betaproteobacteria  Cyanobacteria  Firmicutes - Clostridia  Planctomycetes  Deltaproteobacteria  Verrucomicrobia  Acidobacteria | 5384-6261 | 5858 | 13 | 18 | 38 | 21 | 11 | 6 | 9 | 4 | *Xanthobacter dioxanivorans* | It is a gram-negative bacterium capable of degrading 1-4-dioxane residue from industry with high toxicity by human health. |
| Actinobacteria  Alphaproteobacteria  Betaproteobacteria  Firmicutes - Bacilli  Gammaproteobacteria - Others  Verrucomicrobia  Bacteroidetes  Cyanobacteria  Gemmatimonadetes  Gammaproteobacteria - Enterobacteria  Planctomycetes  Deltaproteobacteria  Acidobacteria | 6262-7139 | 6710 | 11 | 18 | 38 | 22 | 10 | 6 | 9 | 4 | *Rhizobium leguminosarum* | It is a soil-inhabiting bacterium that has shown an effective N2-fixing principally with function as a microsymbiont of Trifolium. |
| Actinobacteria  Alphaproteobacteria  Betaproteobacteria  Firmicutes - Bacilli  Gammaproteobacteria - Others  Verrucomicrobia  Bacteroidetes  Cyanobacteria  Gemmatimonadetes  Gammaproteobacteria - Enterobacteria  Planctomycetes  Deltaproteobacteria  Acidobacteria | 7140-8017 | 7571 | 10 | 18 | 38 | 22 | 10 | 5 | 9 | 3 | *Nocardia nova* | It has degrading enzymes of polyisoprenoids, a rigid and degradation resistant material commonly used in wide range applications. |
| Deltaproteobacteria  Actinobacteria  Betaproteobacteria  Alphaproteobacteria  Bacteroidetes  Cyanobacteria  Planctomycetes  Firmicutes - Bacilli  Gammaproteobacteria - Others  Acidobacteria | 8018-8895 | 8538 | 10 | 16 | 39 | 24 | 10 | 5 | 9 | 3 | *Actinoplanes friuliensis* | This microorganism is a gram-positive bacterium capable of producing antibiotic friuli mycin, which is active against *Staphylococcus or Enterococcus*. |
| Actinobacteria  Deltaproteobacteria  Alphaproteobacteria  Betaproteobacteria  Chloroflexi | 8896-9773 | 9372 | 9 | 16 | 40 | 23 | 10 | 5 | 10 | 3 | *Sorangium cellulosum* | It is an obligate aerobe bacterium capable of synthesizes cytotoxic chivosazoles and catecholate type siderophores myxochelins, which has inhibitor activity of lipoxygenases expressed in immune, epithelial, and tumor cells. |
| Actinobacteria  Deltaproteobacteria  Cyanobacteria | 9774-10651 | 10218 | 9 | 14 | 45 | 23 | 9 | 5 | 8 | 3 | *Kbdelosporangium phytohabitans* | It is member of genus *Kibdelosporangium* important producers of new antibiotics with glycopeptide, macrolides and polyketides structures. |
| Cyanobacteria  Actinobacteria  Deltaproteobacteria | 10652-11529 | 11160 | 8 | 14 | 43 | 23 | 8 | 5 | 9 | 3 | *Nostoc flagelliforme* | It is a filamentous blue-green alga that lives in terrestrial and aquatic habitats which is considered a human food source. |
